# MmpL3 Inhibition: A New Approach to Treat Nontuberculous Mycobacterial Infections

**DOI:** 10.3390/ijms21176202

**Published:** 2020-08-27

**Authors:** Jigar P. Sethiya, Melanie A. Sowards, Mary Jackson, Elton Jeffrey North

**Affiliations:** 1 Department of Pharmacy Sciences, School of Pharmacy & Health Professions, Creighton University, Omaha, NE 68178, USA; jigarparassethiya@creighton.edu (J.P.S.); melaniesowards@creighton.edu (M.A.S.); 2 Mycobacteria Research Laboratories, Department of Microbiology, Immunology and Pathology, Colorado State University, Fort Collins, CO 80523, USA; mary.jackson@colostate.edu

**Keywords:** nontuberculous mycobacteria, mycolic acid, MmpL3, *Mycobacterium abscessus*, susceptibility reversion

## Abstract

Outside of *Mycobacterium tuberculosis* and *Mycobacterium leprae*, nontuberculous mycobacteria (NTM) are environmental mycobacteria (>190 species) and are classified as slow- or rapid-growing mycobacteria. Infections caused by NTM show an increased incidence in immunocompromised patients and patients with underlying structural lung disease. The true global prevalence of NTM infections remains unknown because many countries do not require mandatory reporting of the infection. This is coupled with a challenging diagnosis and identification of the species. Current therapies for treatment of NTM infections require multidrug regimens for a minimum of 18 months and are associated with serious adverse reactions, infection relapse, and high reinfection rates, necessitating discovery of novel antimycobacterial agents. Robust drug discovery processes have discovered inhibitors targeting mycobacterial membrane protein large 3 (MmpL3), a protein responsible for translocating mycolic acids from the inner membrane to periplasm in the biosynthesis of the mycobacterial cell membrane. This review focuses on promising new chemical scaffolds that inhibit MmpL3 function and represent interesting and promising putative drug candidates for the treatment of NTM infections. Additionally, agents (FS-1, SMARt-420, C10) that promote reversion of drug resistance are also reviewed.

## 1. Introduction

Nontuberculous mycobacteria (NTM) are mycobacteria, other than *Mycobacterium tuberculosis (M. tb)* and *Mycobacterium leprae*, the causative agent for tuberculosis (TB) and leprosy, respectively, that are environmental pathogens and are commonly found in soil, dust, biofilms, and natural and municipal water sources. NTM are also described in literature as atypical mycobacteria or mycobacteria other than tuberculosis (MOTT) [1]. To date, approximately 200 NTM species have been identified. Depending on the growth rate, NTM species are classified into rapid growing mycobacteria (RGM) and slow growing mycobacteria (SGM). The RGM includes *Mycobacterium abscessus* complex (MABSC)*, M. chelonae, M. fortuitum, M. smegmatis,* and *M. vaccae*, whereas SGM include *M. avium complex* (MAC)*, M. kansasii, M. xenopi, M. marinum, M. simiae, M. haemophilum, M. ulcerans,* and *M. gordonae*. MAC includes *M. avium, M. intracellulare,* and *M. chimaera*, whereas MABSC includes *M. abscessus* subsp. *abscessus, M. abscessus* subsp. *bolletii,* and *M. abscessus* subsp. *Massiliense*. *M. abscessus* is establishing itself as a highly pathogenic bacteria and a recent review was published describing mycobacterial morphotypes and their role in the pathogenesis and current strategies against *M. abscessus* [2,3].

NTM infections are mostly seen in immunocompromised patients or those with underlying structural lung diseases such as cystic fibrosis (CF), bronchiectasis, chronic obstructive pulmonary disease (COPD), pneumoconiosis, prior TB, pulmonary alveolar proteinosis, and esophageal motility disorders [4,5]. Although NTM-pulmonary disorders (NTM-PD) are most commonly observed with NTM infections, extrapulmonary infections of the skin and soft tissues, lymph nodes, and blood have also been confirmed. Person-to-person transmission of NTM infections has only been documented in CF patients with *M. abscessus* pulmonary infections [1]. The species that most commonly cause NTM-PD are MAC*, M. kansasii, M. abscessus,* and *M. xenopi* [6].

Over the past several decades, the incidence and prevalence of NTM infections have been steadily increasing [7,8]. Unlike TB, it is difficult to determine the worldwide prevalence of NTM infections, as it is not mandatory to report in most countries. Therefore, the global prevalence of NTM infections remains unknown, and is likely underreported [7,9,10]. Moreover, imprecise follow-up, improper diagnosis or unavailability of tests, and difficulty in differentiation between infection and disease are other challenges faced in the diagnosis and treatment of NTM infections [7]. NTM infections are often misdiagnosed as TB leading to mistreatment, longer hospitalization, and waste of resources/drugs [5]. For many years, *M. abscessus* was misdiagnosed with *M. chelonae*, which rarely causes NTM-PD [11]. CF patients are at significantly increased risk of NTM infections, with incidence rates of 3.3–22.6% [12], and COPD patients on chronic corticosteroid therapy have been found to have a 29-fold increased risk of NTM infection [13]. On 12 May 2020, the World Health Organization (WHO) published an information note on TB and COVID-19 (a new coronavirus disease 2019). Projections in this note estimate an additional 1.4 million TB deaths (13% rise in TB deaths) in the next 5 years due to the COVID-19 pandemic [14]. This estimate raises concern about the possibility of increased incidence of NTM-associated morbidity and mortality in coming years.

In 2007, the American Thoracic Society (ATS) and the Infectious Diseases Society of America (IDSA) published an official guideline for the diagnosis, treatment, and prevention of NTM infections which has been recently updated in 2020 [6,15]. The standard treatment regimen for majority of NTM species is a triple antibiotic regimen for at least 18 months or 12 months after a first negative culture [15], NTM-PD caused by *M. kansasii* are among the easiest to treat, as it is susceptible to traditional antitubercular agents, excluding pyrazinamide [16]. Regimens utilized in the treatment of MAC infections typically include a macrolide, including clarithromycin or azithromycin, in addition to rifampicin and ethambutol [6]. The current recommended therapy for MABSC infections is a macrolide with concurrent parenteral intravenous antibiotics (amikacin, cefoxitin, imipenem, or tigecycline) for 2 to 4 months, followed by step-down therapy with a macrolide and another oral or inhaled antibiotic agent (linezolid, clofazimine, or inhaled amikacin) [6]. However, treatment outcomes remain poor [5], and the extended multi-drug regimens contribute to significant major adverse reactions [6]. Furthermore, in the 3 to 4 years subsequent to antibiotic therapy, relapse rates range from an alarming 25 to 50% [17,18,19]. Of all NTM infections, NTM-PD caused by *M. abscessus* subsp. *abscessus* remains the most difficult to treat, with average treatment success rates below 50% [20,21,22]. Furthermore, in CF patients with confirmed *M. abscessus* isolates, lung transplantation is considered relatively contraindicated because of the high incidence of poor patient outcomes [23]. Therefore, infections from MABSC are considered “incurable nightmares” [5], and NTM species the “New Uber-Bugs” [24].

**Figure 1:**
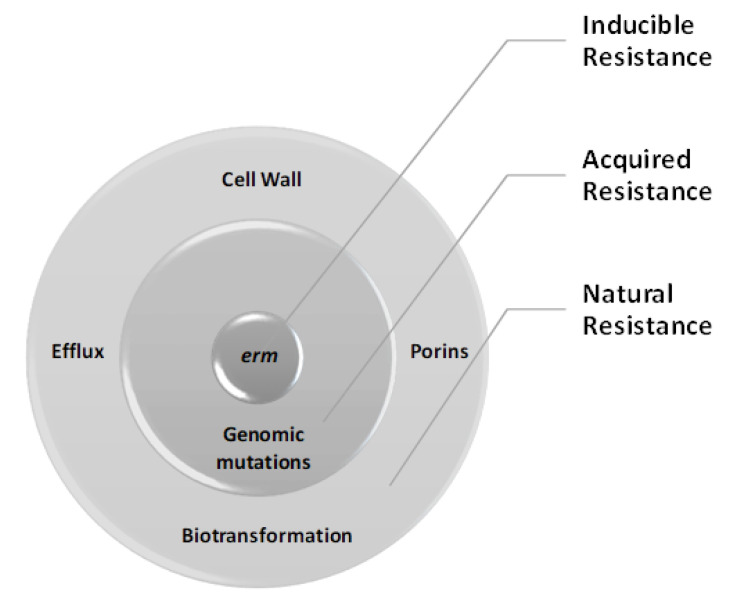
Mechanism of resistance to the nontuberculous mycobacteria.

NTM species are highly resistant to antibiotics, sterilizing agents, antiseptics, and disinfectants [25]. NTM show resistance to antibiotics through natural resistance, inducible resistance, and mutational resistance (Figure 1). The most common natural resistance is reduced cell envelope permeability. The highly lipophilic outer membrane of the cell envelope blocks the penetration of many antibiotics. Several genes such as protein kinase G (*pknG), fbpA, asnB, kasB, mtrAB, pks12, Maa250,* and *mspA* porin promotes multi-drug resistance. Furthermore, MAC is known to overexpress genes encoding efflux pumps, including *P55, mmpl5, tetV, tap, efpA,* and *lfrA*, which increases drug resistance due to reduced exposure to antibiotic agents. Another mechanism of natural resistance is biotransformational deactivation of drugs via enzyme-mediate catalysis, which has reduced the effectiveness of rifampicin, quinolones, beta-lactams, and aminoglycosides. Inducible resistance occurs through induced expression of erythromycin resistance methylase (*erm*) genes, which result in the translation of a methylase that weakens the binding of macrolides with the bacterial ribosomes. Acquired resistance by mutations in the 23S rRNA (*rrl*), 16S rRNA (*rrs)*, and *rpoB* genes of the NTM species causes high-level resistance to the macrolides and linezolid, aminoglycosides, and rifampicin, respectively. Bedaquiline also shows acquired resistance against MAC pulmonary disease through the regulator gene of the MmpS5/MmpL5 efflux system, and this mutation results in cross-resistance to clofazimine [26].

The increasing incidence, limited efficacy of current treatment options, and significant risk of drug-resistance in NTM infections suggest an urgent need for new antimycobacterial agents. While several drug candidates, such as inhaled nitric oxide gas, gallium nitrate, interferons, IL-12, clofazimine, bedaquiline, rifabutin, amithiozone, linezolid, tedizolid, and tigecycline, are under clinical trials for NTM infections, most of them are repurposed antibiotics drugs used for TB or other bacteria. The drug discovery pipeline is primarily focused on efficacy against TB, with few trials assessing agents effective for treatment of NTM infections. Recently developed compounds targeting the MmpL3 transporter, a new and promiscuous target recognizing many structurally unique chemotypes, have proven to be effective in both TB and NTM infections. In this review, we focus on potential new compounds that inhibit MmpL3 with efficacy against NTM species.

## 2. Mycolic Acid Biosynthesis

The mycobacterial cell wall is different from the Gram-positive and -negative bacterial cell wall [27]. It is a complex structure, made up of covalently linked mycolic acids (MA)-peptidoglycan (PG)-arabinogalactan (AG) (also known as mAGP) [28]. The peptidoglycan is the innermost layer and mycolic acids are the outermost layer of the mycobacterial cell wall [28]. Mycolic acids are a major component and are considered a hallmark of the mycobacterial cell wall [27,29,30]. Mycolic acids comprise a highly impermeable lipid-rich layer that protects the mycobacterial cell against various threats including antibiotics and the host’s immune system and also contributes to virulence [30,31]. Chemically, mycolic acids are α-alkylalted, β-hydroxylated long-chain fatty acids. The α-alkyl chain comprises saturated C_22_-C_26_ carbons, and the β-hydroxy long meromycoloyl chain comprises C_42_-C_62_ carbons [32]. Depending on the functional groups attached, the mycolic acids found in *M. tb* can be differentiated into α-, methoxy-, keto-, and/or hydroxy-mycolic acids. There are in total C_66_-C_90_ carbons in a mycolic acid chain length [27,30]. Even though mycolic acids are found in all mycobacterial pathogens and provide similar cellular protection, structural integrity, and virulence, subtle differences in carbon chain length an composition are found. For example, at the time when this review was written, keto- and methoxy-MA have not been detected in *M. phlei*, [29], yet are common in *M. tb*. These structural differences may account for the variations in drug susceptibility among mycobacterial pathogens.

**Figure 2:**
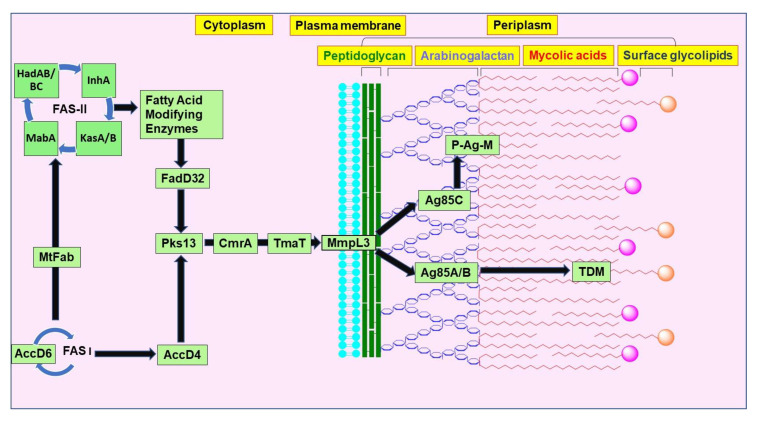
Pictorial representation of key enzymes, transporters and transferases involved in the mycolic acid biosynthetic pathway. β-ketoacyl-ACP synthase A (KasA), β-ketoacyl-ACP synthase B (KasB), β-ketoacyl-ACP reductase (MabA), β-hydroxyacyl-ACP dehydratase (Had), enoyl-ACP reductase (InhA), MtFab, acyl-CoA carboxylase (AccD), fatty acid adenylating enzyme (FadD32), polyketide synthase 13 (Pks13), *Cg1* acetyltransferase (TmaT), mycobacterial membrane protein large (MmpL3), mycolyltransferases (Ag85 complex), trehalose monomycolate (TMM).

The biosynthetic pathway of mycolic acids involves many catalytic enzymes that synthesize and functionalize long fatty chains that are condensed together, transported out and attached to the outer membrane (Figure 2). The biosynthesis of mycolic acids initially involves two enzyme complexes: fatty-acid synthase-I (FAS-I) and fatty-acid synthase-II (FAS-II). FAS-I initiates de novo fatty acid synthesis cycle from an acetyl group to produce C_16-18_ and C_24-26_ acyl CoAs [29]. Each cycle of FAS-I synthase undergoes the addition of two new carbon atoms, thereby increasing the chain length [30]. FAS-II further elongates the short chain C_12-16_ fatty acids to C_18-30_ acyl-acyl carrier proteins (ACPs) [29]. FAS-I synthesizes hexacosanoyl-CoA (C_26_) that ultimately becomes the α-chain of mycolic acids [30]. β-Ketoacyl-ACP-synthase III (MtFabH) initiates the fatty acid synthesis by combining malonyl-ACP and acyl-CoA through Claisen condensation. The product formed is β-ketoacyl-ACP, followed by a reduction to β-hydroxyacyl-ACP by β-ketoacyl-ACP reductase (MabA). β-Hydroxyacyl-ACP dehydratases (HadAB and HadBC) convert the β-hydroxyacyl-ACP into an enoyl-ACP. This enoyl-ACP is reduced to an acyl-ACP by NADH-dependent trans-2-enoyl-ACP reductase (InhA). This acyl-ACP is again taken up into the FAS-II cycle and further elongated until a mero-mycolic acid chain is formed. It is thought to undergo five cycles for alpha-mycolic acids and eight cycles for methoxy- and keto-mycolic acid. Elongation of the fatty acid chain takes place by β-ketoacyl-ACP-synthase (KasA or KasB). Marrakchi et al. report InhA, MabA, HadB, and KasA as essential proteins [29]. In addition, KasB is essential for the full extension of the mycolic acids, the acid-fastness property of the mycobacteria, and production of ketomycolic acids [27,33]. The addition of cyclopropanes to the meromycolic acid is catalyzed by the S-adenosyl-methionine (SAM)-dependent methyltransferases. CmaA1, CmaA2, PcaA, and MmaA2 are the methyltransferases identified in *M. tb* [29], however, the exact NTM enzymes have not been determined. The product from the FAS-I is carboxylated by AccD4 (acyl-CoA carboxylase) [29], as AccD4 and AccD6 are essential genes in *M. tb* [34]. Before the final condensation with the short α-chain, the long-chain meromycolic acid (C_50-60_) gets activated through the FadD32 enzyme. FadD32 (fatty acid adenylating enzyme) is a member of the fatty acyl-AMP ligase (FAAL) class that activates and transfers the activated long chain acyl adenylate to the pantetheine moiety of the N-terminal acyl carrier protein (ACP) domain of polyketide synthase 13 (Pks13) [29,35]. Pks13 is a member of the type-I Pks family and is responsible for performing the final condensation step in the production of mycolic acids. The five domains of the Pks13 are peptidyl carrier protein (PCP)-like domain, ketoacyl synthase (KS), acyl transferase (AT), ACP domain, and thioesterase (TE). 4′-Phosphopantetheinyl transferase PptT is essential for the activation of the ACP and PCP domains of Pks13 [29]. After the final condensation step, a reduction mediated through CmrA produces mature mycolic acids and their Pks13-mediated transfer to trehalose to form trehalose monomycolate (TMM) [29]. After acetylation of the β-hydroxy group on TMM by TmaT, TMM is transported out of the cytoplasm to the periplasmic space through the MmpL3 transporter [36,37]. Subsequently, this glycolipid serves as a donor of mycolic acyl chains that either form a covalent linkage with the arabinogalactan layer of mAGP or esterify another molecule of TMM to form the outer membrane glycolipid, trehalose dimycolate (TDM, also known as cord factor) [38,39]. The enzyme responsible for the transesterification of mycolic acids from TMM to their cell envelope acceptors are the mycolyltransferases (Ag85 complex), FbpA, FbpB, and FbpC (also known as Ag85A, Ag85B, and Ag85C). Ag85C helps in the formation of mAGP whereas Ag85A and Ag85B help in the formation of TDM [32]. Although TDM is present mainly as the outer layer, phthiocerol dimycocerosate (PDIM) and a number of other acyltrehaloses (sulfolipids, diacyl-, and poly-acyltrehaloses) also contribute to form an outer layer [39].

### 2.1. MmpL3 as a Promiscuous Target

**Figure 3:**
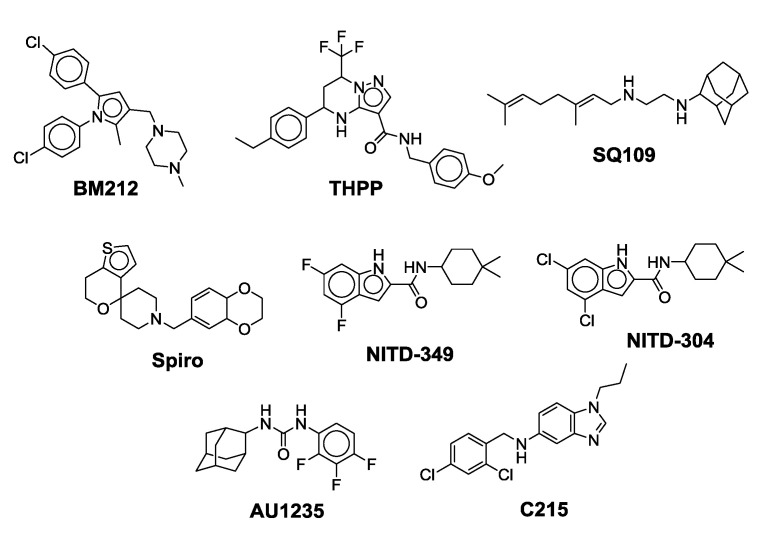
Lead preclinical and clinical MmpL3 inhibitors.

MmpL3 transporters aid in the translocation of TMM across the plasma membrane for cell envelope biosynthesis. MmpL3 belongs to the resistance-nodulation-cell division (RND) protein superfamily of inner membrane transporters. Its translocation activity is dependent on the proton motive force (PMF). On the basis of a spheroplast-based translocation assay that can determine the topology of TMM in the plasma membrane, Xu and colleagues proposed that MmpL3 functions as a TMM flippase [40]. In the MmpL family, MmpL3 is an essential MmpL transporter indicating that its function is unique. Co-crystallography and native mass spectrometry studies indicated that MmpL3 not only binds TMM but also phospholipids such as phosphatidylethanolamine, phosphatidylglycerol, and cardiolipin suggesting that it might be involved in the translocation of more than one lipid to the periplamic space [39]. Determination of the crystal structure of MmpL3 from *M. smegmatis* indicates that MmpL3 functions as a monomer [39,41]. Although this observation is contradictory to earlier gel filtration and single-particle electron microscopy studies indicating that MmpL3 and its ortholog in *Corynebacterium glutamicum* (CmpL1) form trimers, the same way prototypical RND transporters from Gram-negative bacteria do [42]. We believe this discrepancy to be due to the fact that the recombinant forms of MmpL3 that were crystallized were devoid of their cytoplasmic C-terminal domain which our preliminary results indicate is required for the proper oligomerization of the transporter [43]. The MmpL3 transporter is essential for the biosynthesis of TDM and the mycolylation of the cell wall arabinogalactan that are required for the replication and viability of mycobacterial cells. Genetic or chemical silencing of MmpL3 leads to rapid cell death in vitro and the same is observed in cellular and in vivo acute infection models [44,45]. Therefore, MmpL3 is considered as an attractive drug target [38,46,47,48]. The MmpL3 ortholog from *M. tb* is 99%, 64%, and 56% identical to the MmpL3 orthologs of *M. bovis*, *M. smegmatis*, and *M. abscessus*, respectively [49]. Inhibition of the MmpL3 transporter results in the accumulation of TMM concentrations intracellularly, and a reduction in the levels of mAGP and TDM [36,37,39,41]. There have been two proposed mechanisms by which an inhibitor can act on MmpL3. The first is that the inhibitor blocks the translocation of the TMM by directly binding to the transporter. The second proposed mechanism is an indirect inhibition of MmpL3 caused by dissipation of the proton motive force (PMF). Several MmpL3 inhibitors such as **AU1235** (adamantyl urea), **SQ109** {N-adamantan-2-yl-N=-[(E)-3,7-dimethyl-octa-2,6-dienyl]-ethane-1,2-diamine}, **BM212** (pyrrole), **THPP** (tetrahydropyrazolo[1,5-a]pyrimidine-3-carboxamide), **Spiro** {N-benzyl-6=,7=-dihydrospiro (piperidine-4,4=-thieno[3,2-c]pyran)}, **C215** ((N-(2,4-dichlorobenzyl)-1-propyl-1H-benzo[d]imidazol-5amine), **NITD-349**, and **NITD-304** (indolcarboxamides) have been described (Figure 3) [50,51,52]. There are no FDA-approved and marketed antibiotics that inhibit MmpL3. However, MmpL3 inhibitors do not share a common pharmacophore, suggesting that MmpL3 is a promiscuous drug target [53]. This article focuses on the new scaffolds of MmpL3 inhibitor found to be active against NTM species.

### 2.2. MmpL3 Inhibitors

#### 2.2.1. Indole Derivatives

Indole-2-carboxamide derivatives are one of the most extensively studied classes of the MmpL3 inhibitors. The compounds of this class show potent action against *M. tb* and NTM species. The NITD compounds (shown in Figure 3) are previously reported indole-2-carboxamides under preclinical evaluation [54]. Structurally, the compound consists of an indole ring at the left-hand side (LHS) and a cycloaliphatic group at the right-hand side (RHS) connected through an amide linkage. The compounds containing indole-2-carboxamide pharmacophore are additionally reported to be effective against drug-susceptible and drug-resistant TB [55].

In 2017, Franz and colleagues reported series of indole-2-carboxamides with activity against NTM species including *M. abscessus, M. bolletti, M. massiliense, M. avium, M. smegmatis*, and *M. cholenae* [56]. The compounds were synthesized into two miniseries: unsubstituted indoles and 4,6-dimethyl substituted indoles. From the first series, adamantyl- and isopinocampheyl-containing unsubstituted indoles show activity against RGM with MIC <1 µg/mL, except *M. smegmatis*. From the second series, the compound with the highest potency contained a cyclooctyl ring and 4,6-dimethyl indole (**1**, Table 1), having an activity ranging from 0.0039 to 0.6 µg/mL against RGM. Compounds with cycloheptyl, isopinocampheyl, and 4-methylcyclohexyl head groups also showed significant activity against mycobacteria. Replacing the head group with an aliphatic long chain, phenyl ring, or surprisingly, adamantyl group rendered the compound ineffective. Compound **1** is also active against SGM *M. avium* with MIC = 0.05–1 µg/mL and *M. xenopi* with MIC = 0.25 µg/mL. In vitro cytotoxicity studies show no toxicity against THP-1 cells and the selectivity index (SI) of >1910 for *M. abscessus*, *M. massiliense*, and *M. bolletii* [56]. In vivo studies with **1** conducted in mice demonstrated good oral bioavailability and were efficacious in *M. abscessus* infected mice [57,58]. Compound **1** was also identified as a screening hit performed by Low and colleagues, where **1** is reported as **MMV687146** with MIC_50_ = 0.8 µg/mL [59].

The SAR was further explored by replacing the 4,6-dimethyl groups with halogen functional groups. As aromatic methyl groups are vulnerable to CYP-mediated benzylic oxidation, replacing them with halogens may result in compounds with higher metabolic stability and similar lipophilicity when compared to previous series [60,61]. From the series of compounds tested, **2**, **3**, and **4** showed activity similar to compound 1 against *M. abscessus*, with MIC values of 0.125 µg/mL. Compound **2** is a 4,6-dichloro substituted indole, whereas **3** is a 4,6-difluoro substituted indole. Compound **4** retains similar activity as a monosubstituted 6-bromo-indole. A 4,6-dibromo-substituted indole was not studied. The bulkier cyclooctyl ring is optimal for the activity against *M. abscessus* [62]. Variation in the amide linkage can result in loss of activity. Linking functionalities including ketoamide, oxamide, 1,1-diamide-amine, and thiazole-2-amide were assessed against *M. tb* but did not improve activity [60].

Compounds **2** and **4** were found to be equally effective against 30 clinical strains including smooth (S) and rough (R) variants isolated from the CF and non-CF patients. Both compounds act by inhibiting TMM transport and lack activity against Ag85 complex enzymes. Compound **2** has the capacity to permeate human macrophages to elicit its action. However, the ADME properties of **2** show low permeability, high plasma protein binding, and high intrinsic clearance. In addition, the MmpL3 A309P mutant shows high resistance to **2** [62].

Compound **5**, a 4-fluoro-n-octyl-3-[(azepan-1-yl)methyl] indole, demonstrates high potency with MIC_50_ values of 2.3 µM, 2 µM, 8 µM, 19 µM, and 2 µM against *M. tb, M. bovis BCG, M. smegmatis, M. abscessus,* and *M. avium*, respectively [63]. This interesting scaffold is thought to act pleiotropically, having multiple other cell membrane-embedded targets, including MmpL3. Li and colleagues found that **5** disrupts the PMF which may contribute to the inhibition of MmpL3. In addition to reducing extracellular levels of TDM, it also decreases the level of other cellular lipids. However, **5** does not have a docking pose with the MmpL3 protein, is not cross-resistant with direct MmpL3 inhibitors, and retains activity against the MmpL3 resistant mutants [64].

Compound **5** is an optimized analog of **SQ109**, 1-geranylindole or (5-fluoro-[(E)-1-(3,7-dimethylocta-2,6-dien-1yl)]-3-(piperidin-1-ylmethyl)-1H-indole) with moderate activity and selectivity (MBC_90_ = 12 µM and IC_50_ Vero = 22 µM). Substitutions at the 1-and 3-positions on **5** are essential to maintain antimycobacterial action. Various side chains have been assessed at the 1-position, such as n-butyl, n-octyl, 3-cyanopropyl, N-substituted aminocarbonylmethyl, benzyl, phenpropyl, phenoxypropyl, and cyclohexylethyl. Optimal action is produced by the n-octyl side chain. Moreover, an increase in chain length up to 12 carbons does not affect the activity. Next, the substitution of the saturated cyclic ring at the third position is important. Replacement of the azepane ring with an aliphatic chain with a tertiary nitrogen decreases the activity. Substitution with cyclopropyl or cyclopentyl causes a small drop in the activity, whereas 4-methylpiperidine and N-methyl cycloheptylamine retain activity. While morpholine, piperazine, and thiazine groups decrease the activity, the 1,4-dioxa-8-azaspiro[4.5]decane (*M. bovis* MIC_90_ = 5 µM) shows higher activity than **5** (*M. bovis* MIC_90_ = 6 µM). Replacing the fluoro group with chloro, bromo, or methoxy maintains the activity. However, the position of the groups correlates with the activity. Azaspiroketals with 6-methoxy substitutions are potent, whereas azapenes with 4-fluoro substitutions are potent compounds [63].

Despite being active against mycobacteria, **5** also has activity against the Gram-positive bacteria *S. aureus* (MIC_50_ = 8 µM), but is not active against the Gram-negative bacteria *E. coli*, suggesting **5** interacts with additional pharmacological targets. However, **5** has some toxicity against HepG2 and Vero cell lines (IC_50_ = 19.2 µM and 29.2 µM, respectively). In vitro studies determined moderate solubility (35.9 µM) and good metabolic stability with a half-life of 560 min tested on rat liver microsomes [63]. Considering the promising activity against *M. avium*, further optimization of this scaffold is warranted to reduce potential for toxicity.

Additionally, the azaspiroketal derivatives were found to be more potent than **5** against *M. tb* and directly bind MmpL3, as supported by cross-resistance with other MmpL3 inhibitors. In vivo pharmacokinetic studies in murine models have been reported, proving promising ADME parameters, and screens against NTM species should be pursued [64,65].

#### 2.2.2. Benzimidazole Derivatives

In 2014, Gobis et al. reported **6** as an antimycobacterial agent with activity against *M. tb* and *M. bovis* [66]. The mechanism of action for this compound was later identified as MmpL3 inhibition [67]. Compound **6** is a 2-(2-cyclohexylethyl)-5,6-dimethyl-1*H*-Benzo[d]-imidazole. Recent in vitro studies of benzimidazole derivatives show bacteriostatic action of **6** against *M. abscessus* clinical isolates including S and R variants from CF and non-CF patients. Unlike the optimal 4,6-disubstitution patterned indole-2-carboxamides, the highly potent compounds from this series are 5,6-disubstituted benzimidazoles. In addition, this series lacks the amide side chain and the bulky cycloaliphatic group such as cyclooctyl, adamantyl which is thought to be a characteristic feature of MmpL3 inhibitors. Despite this, **6** has similar activity to that of previously reported indoles **2**, **3**, and **4** (MIC = 0.125 µg/mL). Similar to indole-2-carboxamides, the NH hydrogen is required to form an H-bond with the MmpL3 transporter. Replacement of the hydrogen with the aryl sulfonyl group results in inactivity. In contrast to indole-2-carboxamide derivatives, mono-substitution at the **6** position of the phenyl ring of benzimidazole significantly decreases the activity. Increasing the length or degree of unsaturation of the chain also decreases the activity. A small mini-series with a phenyl group replacing the cyclohexyl group on the RHS was also assessed. All compounds within the series were inactive except one with a 3,5-dichlorophenyl having the MIC = 0.25 µg/mL [68].

Benzimidazole derivatives show acceptable cytotoxicity with SI of 712 against THP-1 macrophages. Compound **6** decreases the intracellular bacterial load in infected macrophages and in embryonic zebrafish models. However, the study reveals that many MmpL3 mutants are resistant to benzimidazole derivatives. In assessing the MmpL3 mutants, the potency drops >64-fold for the A309P mutant while a 4- to 8-fold drop is seen for the other mutants. However, greater resistance was seen against **AU1235** than for **6**. Of note, **2** (indole-2-carboxamide) is effective against all MmpL3 mutants, except the A309P mutant. Compound **6** is cross-resistant with compound 13 (piperidine derivative) but not with SQ109. It is assumed that **6** acts by direct MmpL3 inhibition, based on the available data. All reported mutants, except V299A, have mutations located far from the binding site of MmpL3. It is thought that mutations in MmpL3 can induce long-distance structural rearrangements that can result in reduced drug-binding affinity [50,68].

William and colleagues identified MmpL3 inhibitors through high-throughput screening. Of the hit compounds, two contain the benzimidazole pharmacophore. Compound **7**, 2-[(5-chloro-1H-benzimidazol-2-yl)sulfanyl]-N,N-di(propan-2-yl) acetamide is the only active benzimidazole and was among the most potent compounds identified in the screen against *M. abscessus*. Compound **18** (4-thiophen-2-yloxane-4-carboxamide analog) was the most potent compound against *M. abscessus* among the hits (described below). Compound **7** contains a sulfur group in the amide side chain and lacks the cycloaliphatic ring system, instead having two isopropyl substituents on the amine. It shows 81.8% growth inhibition for *M. abscessus* at the single concentration of 20 µM (MIC_50_ = 25µM). The compound is selective against the mycobacteria species and lacks activity against Gram-positive and Gram-negative bacteria (>200 µM). Compound **7** has been shown to disrupt the membrane potential, thereby, suggesting its activity may be through disruption of PMF. The bone marrow murine macrophage cytotoxicity study reveals it as a safe compound (CC_50_ > 100 µM). Additionally, **7** has good solubility at 178 µM and good microsomal stability of 71% remaining 30 min after administration. Benzimidazoles with a 5-methyl instead of a 5-chloro group lack activity against *M. abscessus*, suggesting the importance of having an electron-withdrawing group on the benzimidazole ring. The methyl group is also susceptible to the CYP-mediated benzylic hydroxylation and resulted in reduced microsomal stability, dropping it from 71% to 25% [48].

Recently, Dal Molin et al. reported additional benzimidazole derivatives, **8** and **9**, as putative antimycobacterial agents targeting the MmpL3 transporter. Using GFP-based high-throughput screening, several hits were identified against *M. tb* and *M. abscessus*. Compound **8** showed an MIC_90_ of 0.98 μM against *M. tb*. The potency for **8** drops to 62.5 μM against the drug-resistant mutants. Compound **9** is cross-resistant to **8**, and similarly, has a higher MIC of >62.5 μM against **8** resistant mutants. Both compounds have an MIC_90_ of 31.25 μM against *M. abscessus*. Compound **8** was also able to significantly reduce mycobacterial load in an infected macrophage. The pharmacophore for **8** and **9** is an aminobenzimidazole, which differs from **6** and **7**. Compound **8** differs from **9** by the substitution on the benzimidazole ring. Compound **8** is 5,6-dimethyl substituted, whereas **9** is 5-chloro,6-methyl substituted. Compound **8** and **9** lack the amide side chain, like **6**, and lack the cycloaliphatic group at the RHS, like **7**. Instead, they each contain a 2-isopropyl-1-aniline ring attached to the 2-benzimidazole. However, **8** shows high toxicity against THP-1 macrophages (15.625 μM) [69]. There is no evidence of how **8** and **9** inhibit MmpL3, direct or indirect. Moreover, there are no reported in vivo pharmacokinetic studies for **8** and **9**. Optimization is required to increase potency against NTM species.

#### 2.2.3. Benzothiazole Derivatives

Graham et al. report **11** as a potent antimycobacterial agent showing activity against *M. abscessus* ATCC 19977, *M. avium* 101, *M. intracellulare* 1956, and *M. tb* H37Rv. The pharmacophore of this benzothiazole derivative is different than the two previously discussed. The NH of the benzimidazole is replaced by a sulfur atom, comprising the benzothiazole aromatic ring system. Moreover, the amide linkage is reversed here such that the NH of the amide is attached to the benzothiazole ring (LHS) instead of the alicyclic ring (RHS) as seen in other MmpL3 inhibitor classes. Compound **11** is an optimized version of the previous hit **10**, which contained a dimethylated adamantyl ring (*M. abscessus* MIC = 1 µg/mL, *M. avium* MIC = 2 µg/mL, *M. tb* MIC = 4 µg/mL). Various cycloaliphatic groups (RHS) were studied by replacing the adamantyl group because the adamantyl ring is lipophilic and may lead to nonspecific binding. This is a common problem for not only MmpL3 inhibitors, but many antimycobacterial drug discovery efforts as the mycobacterial cell wall is lipid-rich with mycolic acids, leading to initial lipophilic hits that can effectively penetrate the cell wall. Out of the screening process, 3, 3, 5-trimethylcyclohexyl group is found to be most effective against *M. abscessus*. Various mono-, di-, and tri-substitution patterns using functional groups such as X, CH_3_, CF_3_, OCF_3_, OCH_3_, SCF_3_, NCH_3_, and OH were assessed on the benzothiazole ring (LHS). High activity against *M. abscessus* was seen in the 5,7 di-substituted and 6 mono-substituted patterns, with halogens such as fluorine and chlorine proving most effective. However, activity is low against other mycobacteria. It was suggested that the 1-methylated cycloaliphatic ring improves activity against RGM and SGM. The 1-methylcycloheptyl derivative shows >530-fold and >8-fold increase in activity against *M. abscessus* and *M. avium* compared to that of the unsubstituted cycloheptyl [70]. The ineffectiveness of the unsubstituted cycloheptyl against *M. abscessus* using CIP 104536 susceptible strain is reported in another study [62]. Compound **11**, containing the 5-methylbicyclo [3.3.1] nonane group, shows highly potent MICs against RGM such as *M. abscessus* ATCC 19977 (0.03 µg/mL), *M. abscessus* subsp. *massiliense* 119 (0.03 µg/mL), *M. chelonae* 93 (0.03 µg/mL), *M. fortuitum* 41 (0.06 µg/mL), *M. peregrinum* ATCC 700686 (0.03 µg/mL). Moreover, it retained activity against SGM such as *M. avium* 101 (2 µg/mL), and *M. intracellulare* 1956 (2 µg/mL), *M. chimaera* 1502055 (1 µg/mL), and *M. tb* H37Rv mc^2^ 6206 (≤0.12 µg/mL). Active MmpL3 inhibitors, must be lipophilic enough to penetrate the cell envelope. Benzothiazole derivatives exhibited bacteriostatic action against *M. abscessus* and have no activity against Gram-positive and -negative bacteria up to 32 µg/mL. In vitro CYP enzyme inhibition studies show that **11** does not inhibit CYP2B6 and CYP3A4 more than 50% at 10 µM, CYP2B6 and CYP3A4 are the potential CYP enzymes for pulmonary delivery. Also, no hemolytic activity was determined. Unsurprisingly, this class of compounds suffers from low solubility (<3 µM) and high plasma protein binding (>95%). An in vivo study of **10** using granulocyte macrophage-colony-stimulated factor knockout (GM-CSF KO) mice established that there is a reduction of 0.64 Log_10_ CFU in comparison to the vehicle group, but there is no significant reduction observed when compared to the untreated group [71]. The pharmacokinetics in a mouse model for one of its derivatives show 75% oral bioavailability and 1.5 L/h/kg plasma clearance after IV administration (10 mg/kg). In vivo screening, pharmacokinetic studies, and development of an inhaled formulation is in progress for this class [70]. However, MIC assays using clinically isolated S and R variants need to be completed.

In 2014, Shah and colleagues report N-arylalkylbenzo[d]thiazole-2-carboxamides as anti-mycobacterial agents. These compounds contain the benzothiazole ring as an aromatic group on the LHS and a substituted aromatic phenyl ring on the RHS. Unlike indole-2-carboxamides, they contain an additional carbon between the NH amide and the aromatic group on the RHS. Most compounds from the series possess a MIC range of 3.125–50 µg/mL against *M. tb*, except two compounds with MICs of 0.78 µg/mL (**12**) and 1.56 µg/mL (not mentioned). Compound **12** has low toxicity on the HEK-293T (Human Embryonic 25 Kidney cell line) with 32.16% inhibition at 50 µg/mL concentration. Surprisingly, even though it has MmpL3 inhibitor characteristics, molecular modeling suggests that it is a HisG inhibitor. Currently, there are no reported studies assessing efficacy of **12** against NTM [72]. Considering the potency of other benzothiazoles, 10 and **11**, **12** should be screened against NTM species and assessed for MmpL3 inhibition.

#### 2.2.4. Piperidine Derivatives

In 2013, Ballell et al. identified 177 potent, non-toxic lead molecules against *M. tb* [73]. Later in 2016, Dupont and colleagues screened the lead molecules against whole-cell *M. abscessus* that lead to the discovery of the **13** (Known as **GSK1985270A** or **PIPD-1**). Structurally, it is a 4-(4-chloro-3-(trifluoromethyl)phenyl)-1-(2methylbenzyl)piperidin-4-ol, belonging to the piperidinol class of compounds. It is the first piperidinol derivative targeting MmpL3 with a unique scaffold. Being bactericidal in action, it has a MIC_99_ of 0.125 µg/mL against *M. abscessus* reference strains and clinical isolates including smooth (S) and rough (R) variants from CF or non-CF patients. The compound retains its activity against all strains whereas current clinically approved drugs vary in efficacy between strains [74]. Low et al. also identified **13** as active against *M. abscessus* through the pathogen box screening, reported as **MMV688846** with MIC_90_ = 1.5 µM. However, it lacks activity against *M. avium* (MIC_90_ > 50 µM) [59]. Compound **13** is ineffective against Gram-positive and Gram-negative bacteria. Ex vivo studies against THP-1 cell macrophages suggest that the compound enters murine and human macrophages, and has potential to decrease intracellular bacterial replication. In vivo zebrafish infection models reveal that **13** significantly decreases the bacterial load in the treated group as compared to the untreated group. However, MAB_4508 mutants show high level resistance to the piperdinol derivatives, decreasing the potency of **13** by 4-to 64-fold. This MmpL3 inhibition decreases TDM formation and AG mycolylation in the periplasm [74].

Recently, the piperidinol class was explored which led to the identification of **14**. The SAR suggests the importance of two aromatic rings, ring A and ring B, on both sides of the piperidinol. The two substituents, -CF_3_ and -Cl, on ring A are essential for activity. Removing or changing the position of one or both substituents will significantly decrease the activity. Similarly, the substitution of the small ortho group on ring B is optimal for the activity. Replacing the -CH_3_ to -Cl on ring B yields **14** with the same potency (MIC_99_ = 0.125 µg/mL), suggesting preference for a lipophilic group. Similarly, ortho- iodine or bromine-containing molecules have similar potency to that of **14**, but replacement with a fluorine substituent shows an eight-fold decrease in activity. This supports the role of steric hindrance at the receptor site, with larger radii atoms being preferred over smaller radii atoms, suggesting a twisted bioactive conformation. Molecular modeling of the piperidinol derivative reveals that the nitrogen of **13** interacts with the carboxylic group of D618 and the hydroxyl of **13** interacts with the hydroxyl of Y219 through hydrogen bonding. Ring B is required to interact with the phenyl ring of F262 and F622 through π-π stacking interactions [75].

In vitro cytotoxicity studies in a Vero cell line show that **14** is safe, achieving selectivity indices (SI) of 400, whereas **13** has a SI of 200 against *M. abscessus*. A preclinical pharmacokinetic study was performed on BALB/c mice with drug administered via intraperitoneal (IP) route. The highest dosage used in the study was 250 mg/kg, which resulted in a 0% survival rate after 48 h. However, for the 100 mg/kg and 50 mg/kg groups, the survival rates increased to 67% and 100% after 96 h, respectively. More pharmacokinetic parameters were assessed, such as clearance rate, volume of distribution, and elimination half-life, none of which differ significantly from the values obtained by Low et al. The clearance rate and peripheral volume of distribution are 6.9 mL/min and 2.0 L, and the elimination half-life is about 3.2 h. Compound **13** has poor oral bioavailability, calculated at less than 1% [59]. Currently, there are ongoing research efforts to improve the metabolic stability and polarity of this class of compound [75].

Recently, Dal Molin and colleagues identified unique piperidine derivatives, **15** and **16**, as MmpL3 inhibitors through whole cell phenotypic HTS. Compound **15** is phenyl urea whereas **16** is 2-(piperidin-1-yl)ethan-1-amine. Compounds **15** and **16** have *M. tb* MIC_90_ values of >62.5 µM and 15.5–31 µM, respectively, against resistant *M. tb* mutants. Compound **15** is inactive (>62.5 µM) and **16** has MIC_90_ of >31.25 µM against *M. abscessus*. Moreover, both compounds are associated with high cytotoxicity (IC_35_ = ≤ 31.25 µM) [69].

#### 2.2.5. 4-Thiophen-2-yloxane-4-carboxamide

Compound **17** (commonly known as **HC2091**) was identified as an antimycobacterial agent through high-throughput screening. Compound **17** shows moderate activity against *M. tb* with half-maximal effective concentration (EC_50_) of 6.4 µM and the MIC_99_ of 19.3 µM [76]. Compound **17** shows 96.5% growth inhibition of *M. abscessus* at the single concentration of 20 µM, exhibiting a MIC_50_ of 6.25 µM. Additionally, **17** is highly selective for *Mycobacterium* species, lacking action against Gram-positive or Gram-negative bacteria or fungi [48]. Chemically, **17** is a N-[2-(4-chlorophenyl)ethyl]-4-thiophen-2-yloxane-4-carboxamide. This class of compounds is thought to directly inhibit MmpL3 [76]. The ethyl phenyl side chain on the NH of amide is optimal for the activity, removal of ethyl linker drastically decreases activity against *M. tb*, except for 4-isopropylphenyl and 4-trifluoromethyl. Replacing the para-chloro with other groups led to loss of activity [76].

In vitro studies show that **17** binds MmpL3 directly without disrupting the membrane potential. There is no cross-resistance observed with SQ109, suggesting different binding interactions with MmpL3. Compound **17** shows a bacteriostatic effect in macrophages and is safe up to 200 µM [76]. Compound **17** has good kinetic solubility of more than 300 µM. However, it exhibits poor microsomal stability, with only 45% remaining after 30 min [48]. It is also reported that the replacement of the thiophene group is warranted as it is susceptible to *in vivo* metabolism [76]. This interesting class of compounds should be further explored, as it shows potent activity against RGM along with desirable physicochemical properties.

#### 2.2.6. Benzofuran Derivatives

Although benzofuran derivatives are ineffective against *M. tb* [58], they possess activity against *M. abscessus* drug susceptible strain CIP 104536 with a MIC of 0.5–1 µg/mL [62]. Structurally, it resembles the indole-2-carboxamide pharmacophore, except with an oxygen instead of a nitrogen in the aromatic ring system. Therefore, this class lacks a potential H-bond donor interaction with MmpL3 which would have been formed by the NH of the indole. As of now, three benzofuran derivatives have been studied against *M. abscessus*. The two potent compounds (**18** and **19**) contains the cyclooctyl group as the cycloaliphatic ring on the RHS (MIC = 0.5 µg/mL). Compound **18** contains 4,6-dimethylbenzofuran whereas **19** contains 5-chlorobenzofuran. The cyclooctyl group is optimal for the activity, as replacement with an adamantyl ring decreases the activity by two-fold, MIC = 1 µg/mL [62]. Currently, there are no cytotoxicity studies or pharmacokinetic data available for this class of compounds.

#### 2.2.7. Quinoline and Quinolone Derivatives

Alsayed et al. screened a mini-series of quinolones and quinoline derivatives and determined them to be MmpL3 inhibitors. Two quinolone derivatives showed putative antimycobacterial activity against *M. tb* with MICs below 10 µg/mL, whereas quinoline derivatives were ineffective. All the quinolone compounds contain mono- or di-substitution on the LHS and the cyclooctyl or 1-adamantyl ring on the RHS. Compound **20** is a 5-bromo-quinolone linked with 1-adamantyl ring through an amide side chain (*M.tb* MIC = 4 µg/mL). Potency decreases by two-fold when the 5-bromo is changed to 7-bromo (*M.tb* MIC = 8 µg/mL), and 5,7-disubstitution drops the potency by four-fold as compared with **20**. Similarly, 6 or 8 mono-substitution or other di-substitutions result in loss of activity. The activity is higher for the quinolones than its tautomer, 4-hydroxyquinoline, suggesting the importance of the NH in the ring system which may form a H-bond with the receptor. In addition, the carboxamide side-chain at the 2-position is essential for activity, as changing from quinoline-2-carboxamide to quinoline-4-carboxamide results in loss of activity. Although **20** is potent among all other quinolones against *M. tb* it lacks activity against SGM *M. avium* and RGM *M. abscessus* (MIC > 159 µM). It exhibits low toxicity against Vero cells (IC_50_ = 39.87 µM) and has a SI value of 4. However, **20** was found to have poor pharmacokinetic parameters, including solubility of 0.007 mg/mL, Caco-2 permeability of 186 × 10^−6^ cm/s, and 98% plasma protein binding [60]. Further optimization is required to obtain activity against NTM species.

In contrast to previous findings that the quinolines are ineffective, Dan Molin and colleagues identified the quinoline derivative, **21**, which was found to be active against *M. tb* having a MIC_90_ of 7.8–15.6 µM. However, activity against *M. abscessus* is minimal (MIC_90_ > 31.25 µM). The scaffold varies from other MmpL3 inhibitors and **20**. The removal of the methoxy group results in complete loss of activity against *M. abscessus*. Unfortunately, **21** is associated with high cytotoxicity in THP-1 (7.8125 µM) [69]. Currently, there is no available data in vivo pharmacokinetics or on how **21** binds MmpL3. Further optimization of the compound to reduce cytotoxicity and improve activity against NTM species is needed.

#### 2.2.8. Naphthalene Derivatives

Naphthalene derivatives were designed and synthesized as bioisosteric replacements of indole derivatives and further expanded the quinoline and quinolone study. Five naphthalene-2-carboxamide derivatives were synthesized and screened in this study. Out of the five compounds synthesized, two unsubstituted naphthalenes (**22** and **23**) were found to have activity against *M. tb*. They are structurally similar to each other, except for the cycloaliphatic ring on the RHS. Compound **22** contains a cyclooctyl group whereas **23** contains an adamantyl group. Adding substitutions to the naphthalene ring system is detrimental for antimycobacterial activity. Compounds **22** and **23** are active against drug-susceptible and MDR *M. tb* (MIC = 2 µg/mL and 6.5–13 µM, respectively), and have a two-fold increased potency against XDR TB strains (3.27–3.55 µM). However, they lack activity against NTM species such as *M. avium* and *M. abscessus* (MIC > 209 µM) [60].

A cytotoxicity study showed no toxicity against Vero cell lines (>227 µM and >419 µM). The molecular docking study performed on the compounds reveals that there is one less H-bond interaction with the MmpL3 receptor as compared to indole-2-carboxamides, suggesting reduced binding affinity. This supports the importance of the NH hydrogen within the indole ring. Despite the fact that naphthalene and quinoline derivatives both lacks the NH hydrogen; activity is high with the naphthalene derivatives. Because of high lipophilicity of the naphthene ring, these compounds are likely to have poor pharmacokinetic properties. The solubilities of **22** and **23** are 0.002 and 0.007 mg/mL. Furthermore, **22** and **23** have Caco-2 permeabilities of 190 and 207 × 10^−6^ cm/s and plasma protein binding of 95% and 97% [60].

Recently, Gajdár et al. reported the 1-hydroxynaphthalene-2-carboxanilides as potent antimycobacterial agents. The study was performed using cyclic voltammetry and activity was determined based on electrochemical potential. Compounds **24** and **25** were found to be effective against *M. marinum* and *M. kansasii* with MICs of 28.8 µM (**24**) and 28.4 µM (**25**), and were determined to have similar activity against *M. tb*. Using spinach chloroplast, the study showed that these compounds inhibit photosynthetic electron transfer (PET). It is thought that these derivatives have a multi-target effect including ATP synthase and cytochrome bc1 with the disruption of the energy state of the cell [77].

#### 2.2.9. Acetamide Derivatives

Acetamides can be formed with the deletion of 3-carbon atom from the indole-2-carboxamide derivatives. Like indole-2-carboxamides, acetamides contain two rings, the aromatic ring on the LHS and the cycloaliphatic on the RHS, linked together with an amide moiety. Shetty et al. were the first to report acetamides as a new class of MmpL3 inhibitors. Using a single-point *M. bovis* BCG screening assay, they identified 16 hits with MICs below 50 µM. Compound **26** was one of these hits, with an MIC_50_ of 8 µM and MIC_90_ of 12.5 µM against *M. bovis* BCG. Compound **26** is also active against RGM including *M. smegmatis* (MIC_90_ = 50 µM), *M. abscessus* ATCC #19977 (MIC_90_ = 12 µM), and the clinical isolate *M. abscessus* Bamboo (MIC_90_ = 25 µM). However, the compound was ineffective against SGM such as *M. avium* ATCC #35717, and Gram-positive *S. aureus* and Gram-negative *E. coli* with MIC_90_ > 100 µM. Compound **26** blocks the MmpL3 flippase causing an intracellular accumulation of TMM.

To explore the SAR of **26**, ten acetamide analogs were synthesized using the hit as the main pharmacophore. Of the ten acetamides, only four compounds showed an increase in activity (MIC_50_ = 3–7 µM), whereas the other six compounds showed a drastic decrease in activity (MIC_50_ = 10–>100 µM). The lipophilic groups on the aromatic ring are essential for activity. Replacing the 4-trifluoromethyl with polar moieties such as cyano or ester groups decreased the activity, while activity is retained or increased with 3,5-dichloro or 3- or 4-trifluoromethoxy. Changing the 3- or 4- trifluoromethoxy to 2- trifluoromethoxy causes inactivity (MIC_50_ > 100 µM). Cycloaliphatic rings such as cyclohexane or cycloheptane (**27**) showed highest potency against *M. tb* (MIC_50_ = 3 µM), suggesting preference for bulkier cycloaliphatic groups. Replacement of 1-cyanocyclopentane with an aliphatic group such as 1-cyano-1-methyl-isopropyl causes only a slight decrease in activity (MIC_50_ = 10 µM) and replacement with 1-cyano-1-methyl-cyclopropyl results in similar activity to that of the **26** (MIC_50_ = 7 µM) [49]. No further SAR was performed to determine the importance of the 1-cyano group on the cycloaliphatic group. This scaffold is interesting for further SAR development to determine if the activity is correlated to quaternary carbon or cycloaliphatic groups.

Cytotoxicity studies on **26** were performed on HepG2 (human liver carcinoma), THP-1 (human monocytic leukemia), and Vero (African green monkey kidney epithelia) cell lines. Results of these studies support low cytotoxicity and SI of >25 µM. Using rat liver microsomes, the half-life of the compound was found to be 28.6 min and the intrinsic clearance rate was 80.7 µl/min/mg of protein. As **26** lacks or possesses less activity as compared to the indole-2-carboxamides, further optimization and screening against NTM species is warranted [49]. Previous screens show that the 1-substituted cyclooctyl ring has the highest potency against *M. abscessus*. We anticipate **27** to potentially display activity against *M. abscessus*.

#### 2.2.10. Pyrrole Derivatives

**BM212** (**28**) is a commonly known as 1,5-diphenyl pyrrole derivative. Compound **28** has shown activity against replicating and non-replicating *M. tb* including MDR-TB strains (MIC = 8 μM). Interestingly, the activity of **28** is greater against nontuberculous mycobacteria (MIC_M.avium_ = 1 μM) than *M. tb* (MIC = 5 μM). Compound **28** shows moderate cytotoxicity in Vero cell lines (IC_50_ = 9.6 μM) and has a SI value of 1.92. A series of compounds (>300) was generated to improve the potency and physicochemical properties and reduce the cytotoxicity including hERG inhibition. Inhibition of the hERG channel is responsible for the cardiotoxicity which was also associated with the spiro analog (Figure 3). Despite good in vivo activity, the pyrrole derivatives suffer from poor solubility and metabolic profile, high plasma binding, and high hERG channel inhibition. The proposed reason is the highly sp^2^ region and easily protonated nitrogen in the structure. A hit-to-lead approach led to the discovery of **29** with improved in vitro and in vivo activity along with good physicochemical properties.

The early SAR suggests that the para-fluorophenyl at the N1 increases potency and reduces toxicity compared to **28**. To maintain activity, lipophilic groups such as fluoro or electron donating groups such methoxyl or methylsulfanyl are required on the C5 position of the phenyl ring. The (thiomorpholin-4-yl) methyl ring has higher activity than the N-methylpyperazinyl group, later replaced with the morpholine group because of the metabolic instability. Adding N-methylpiperazino-methyl or (thiomorpholin-4-yl) methyl group at the C4 position was found to be detrimental to activity. The para-isopropyl phenyl at C5 showed the highest microsomal stability in the mouse model. The SAR was further studied by removing or replacing N1- and C5-phenyl with alkyl or cycloalkyl in order to decrease the sp^2^ character. Surprisingly, alkyl substituents at the N1 position retain antimycobacterial action. Therefore, new SAR indicates only one phenyl ring at C5 is essential for the activity whereas the N1 phenyl can be replaced with an alkyl or cycloalkyl substituent. The morpholine group is optimal for activity at the C3 position [78,79].

Compound **29** has an isopropyl group at the N1 position and para-isopropylphenyl group at the C5 position along with C2-methyl and C3-methyl morpholine. The compound has a MIC value of 0.15 μM and intramacrophagic MIC of 0.16 μM against *M. tb*. The cytotoxicity study on HepG2 gave To × 50 of 20 μM with a SI of 133. The studied pharmacokinetic properties include membrane permeability of 7.2 × 10^−5^ cm/s, 94.11% HSA binding, and CLND solubility of 199 μM. However, optimization is required to reduce the cytotoxic hERG inhibition (16 μM). Moreover, in vivo study report poor absorption and low bioavailability (F = 1.2%). Compound **29** has moderate systemic clearance value of 16.9 mL/min/kg and a half-life of 1.7 h. At a dose of 50 mg/kg, **29** shows 1.5 log CFU reduction after 9 days of infection [79]. Many pyrrole derivatives from the series have submicromolar activity against *M. tb*. Ultimately, only **28** has shown potent activity against *M. avium*. Given the medical need, the screening of pyrrole derivatives against MAC and other NTM species is needed.

## 3. Susceptibility Reversion

An inhaled liposomal amikacin inhalation suspension (ARIKACYE^®^) has recently been approved by the FDA for the treatment of MAC infections of the lung in adults with limited or no alternative treatment options. This formulation is part of a combination regimen in patients who fail to achieve negative sputum cultures after a minimum of six consecutive months of first-line multidrug antibiotic therapy. The treatment is associated with many adverse effects, with nephrotoxicity being the most serious [81]. In addition, greater than a two-fold drop in potency has been observed in resistant strains of MAC [82,83]. Combining antibiotics should be done cautiously, as some combinations result in synergistic activity and suppression of antimycobacterial resistance, whereas other combinations exhibit deleterious drug–drug interaction.

In vitro synergistic action is observed with amikacin or clarithromycin when administered with clofazimine, and here, it also prevents the re-growth of *M. abscessus* and *M. avium* [84]. Similarly, in vitro studies report an increase in the action of clarithromycin against *M. avium* when combined with tigecycline. Moreover, the combination therapy with tigecycline inhibits development of clarithromycin resistance [85]. Synergistic action against *M. abscessus* is seen when either clarithromycin or tigecycline is co-administered with rifabutin [86]. Rifabutin is also known to inhibit the inducible resistance of clarithromycin in *M. abscessus* [87]. Conversely, in vitro negative drug-drug interaction is observed with the combination of bedaquiline and beta-lactams such as imipenem and cefoxitin [88].

In addition, there are few agents such as **FS-1**, **SMARt-420**, and **C10** that promote reversion of drug resistance and restore the sensitivity of antibiotics.

### 3.1. FS-1

**FS-1** is an anti-MDR/XDR TB drug, approved in 2015 in Kazakhstan [89]. **FS-1** consists of synthesized polysaccharide micellar units containing triiodide complexes coordinated by metal ions and integrated into a dextrin-polypeptide moiety resulting in a 30–300 kDa complex [89]. The polysaccharides act as solubilizing agents and aid in the release of free iodine when mixed with water [90]. The released free iodine causes oxidative stress in the bacterial cell, a mechanism that drug-resistant mutants are more susceptible to [89,91].

**FS-1** induces reversion of drug resistance in MDR- and XDR-TB isolates by making them more susceptible to the oxidative stress induced by **FS-1**. **FS-1** creates high oxidative stress inside the bacterial cell and resistance mutations are more sensitive to the oxidative stress. This led to the reduction in the drug resistance mutations. The reversion of drug resistance to aminoglycosides such as amikacin, kanamycin, and capreomycin has been observed in MDR-TB strains. Adding **FS-1** to the multi-drug regimen of TB prevents the accumulation of new drug resistance mutations and reduces the initial level of drug resistance in MDR-TB infections, and can reduce the duration of MDR-TB treatment from 24 months to 6–12 months [91].

The MIC against the XRD-TB *M. tb* strain SCAID 187.0 is decreased from 27.7 ± 2.4 µg/mL to 21.1 ± 2.0 µg/mL after the administration of **FS-1** for 60 days. Surprisingly, available data suggest that clinical XDR-TB isolates are more sensitive than the drug sensitive *M. tb* H37Rv. In vitro studies of **FS-1** against XDR-TB strain report >2-fold decrease in the MICs for rifampicin, ethionamide, and pyrazinamide, followed by a small MIC decrease for streptomycin and no change in MIC for isoniazid. Like in vitro, in vivo studies using Hartley guinea pigs showed increases in susceptibility to antibiotics against *M. tb* isolates after treatment for 45 days. **FS-1** induces dose-dependent drug resistance reversion with no direct interactions between **FS-1** and the antibiotics. **FS-1** also prevents recurrence of infection, which is commonly observed with antibiotics within 30 days of recovery time [89]. Studies in human lymphocytes and tumor cell lines (HeLa and Caco2) show no DNA damaging action [90]. Moreover, it has been observed in animal models that **FS-1** reduces the hepatotoxicity caused by some antimycobacterial agents [89].

### 3.2. SMARt-420

Biotransformation of the antibiotics causes rapid inactivation of antibiotics via enzymes such as beta-lactamases. On the other hand, biotransformation is also required for pro-antibiotics (prodrug antibiotics) for activation. Mutations in the bioactivating enzymes makes the drug ineffective. For example, mutations in KatG, PncA, and EthA is observed in isoniazid (INH), pyrazinamide (PZA), and ethionamide (ETH)-resistant clinical isolates, respectively [92].

**Figure 4:**
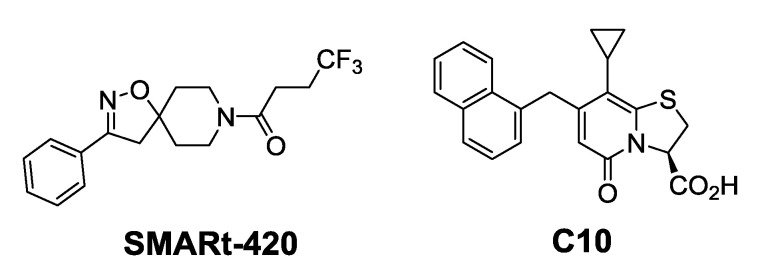
Chemical structures of SMARt-420 and C10.

Blondiaux et al. report Small Molecules Aborting Resistance (**SMARt-420**), a spiroisoxazoline compound, which restores the sensitivity of all ETH-resistant, MDR- and XDR-TB clinical isolates (Figure 4). **SMARt-420** provides an alternative bioactivation pathway to ETH, but does not elicit any antibacterial action. Although ineffective in the absence of ETH, **SMARt-420** boosts the activity of ETH against ETH-sensitive and -resistant *M. tb*. In vivo efficacy studies in a murine model confirm that ETH alone is ineffective against ETH-resistant strains, whereas the combination of ETH with **SMARt-420** significantly reduces the bacterial load (4.6 log) in mouse lungs in a dose-dependent manner. The study proposed that **SMARt-420** triggers cryptic drug-bioactivation pathways in drug-resistant pathogens [92].

### 3.3. C10

**C10** inhibits *M. tb* pellicle formation (MIC = 6.25 µM) and blocks hypoxia-induced tolerance to oxidative stress, acid stress, and isoniazid (INH) (Figure 4). Resistance against INH, a prodrug activated by *KatG* enzymes, typically emerges as a result of mutations in *katG*. **C10** has been found to reverse resistance to INH in *M. tb KatG* mutants. **C10** does not have activity over other antibiotics such as ethambutol, rifampicin, or streptomycin. Moreover, C10 acts synergistically with **Q203**, an ETC inhibitor targeting cytochrome bc_1_ which is undergoing clinical trials [93].

These examples provide insight that drug resistance is not irrevocable and discovery of small molecules could reverse it. Further studies to investigate additional compounds that reverse drug resistance and evaluate use of such agents in combination with antibiotics against drug-resistant NTM strains are warranted.

## 4. Conclusions

The current treatment for the NTM infection includes three antibiotics for at least for 18 months or 12 months after the first negative culture. The regimen includes a combination of approved TB drugs, macrolides, and aminoglycosides. However, treatment outcomes are often poor, the medications used are associated with serious adverse reactions, and there is a high chance of relapse or reinfection to the cured patient. In addition, NTM species are rapidly acquiring resistance to currently available antibiotic options, with resistance being observed even in recently approved drugs [69]. The liposomal amikacin inhalation suspension was recently approved for the treatment of *M. avium* infections. However, the resistance to the amikacin causes drop in the potency by more than two-fold [82]. Therefore, new drugs with novel mechanisms are urgently needed. Recently, many compounds have been developed that show activity against TB and NTM infections that target the MmpL3. As MmpL3 inhibitors act at the level of the plasma membrane, it is possible that some if not all of them can avoid biotransformation by cytoplasmic enzymes.

MmpL3 is an attractive and promiscuous target for TB drugs that plays an important role in cell envelope biosynthesis. TMM, the mycolic acid donor, is synthesized within the bacterial cell and translocated into the periplasm via a mechanism involving the MmpL3 transporter. MmpL3 utilizes the proton motive force to facilitate this translocation. Inhibition of MmpL3 transporter increases TMM levels inside the cell and decreases mycolic acids in the periplasm, thus exerting a bactericidal effect. As mycolic acid biosynthesis is required for the viability in all mycobacteria, MmpL3 inhibitors are effective against both *M. tb* and NTM species. All inhibitors whose interaction with MmpL3 has been characterized to date appear to inhibit MmpL3 directly although some of them display additional disrupting activity on the PMF that may further potentiate their activity against the transporter and confer upon them activity against non-replicating bacilli [50].

The classes of MmpL3 inhibitors explored against NTM species are indoles, benzimidazoles, benzothiazoles, quinolines and quinolones, benzofurans, thiophenes, naphthalenes, acetamides, and piperidines. As per Table 1, Indole-2-carboxamides (**1**, **2**, **3**, **4**), benzimidazole (**6**), benzothiazole (**12**), piperidinol (**13**, **14**), benzofuran (**18**, **19**) show sub micromolar activity against *M. abscessus.* Compounds **1**, **5**, and **12** must be explored further to determine the activity against *M. avium*. Therefore, in vivo testing should be conducted on these scaffolds to further screen against other NTM species. Indole-2-carboxamides are extensively explored via in vitro and in vivo studies and show promising results. However, poor physiochemical and pharmacokinetic properties remain a barrier to drug development. The challenge that most MmpL3 inhibitors face is low solubility due to high lipophilicity. Compounds **7** and **17** show a good solubility with moderate activity, but **7** has high metabolic stability, in contrast to **17**.

Other emerging novel targets such as Pks13 or FadD32, enzymes involved in the mycolic acid biosynthesis, have proven effective against *M. tb*. However, not many compounds have been screened against NTM species. Classes of Pks13 inhibitors include thiophenes, benzofurans, beta-lactams, and coumestans [94]. Despite thiophene (TP2) derivatives having high potency against lab and clinical strains of *M. tb* with MIC = 1 µM , they are inactive against SGM and RGM (MIC > 128 µM) [95]. Similarly, Pks13 inhibitors identified as hits have MIC <1 µM against *M. tb* but >31.25 µM against *M. abscessus* [69], which may explain the lack of screening attempts against other NTM species. Conversely, diarylcoumarins, a class of FadD32 inhibitors, have potent action against SGM (MIC of 0.83 µM and 2.4 µM against *M. marinum* and *M. intracellulare*, respectively) [96]. Recently, a new class of FadD32 inhibitor, the quinolines, were reported to maintain potent antimycobacterial activity while displaying improved pharmacokinetic properties [97]. However, screening of these compounds against the NTM species has not yet been reported.

In addition, **FS-1** and **SMARt-420** could be potential agents to reverse the susceptibility of the drug-resistant NTM species. Both compounds show reversion of drug resistance in the MDR-TB and XDR-TB when co-administered with TB antibiotics. **FS-1** significantly decreases MICs of rifampicin, ethionamide, and pyrazinamide. Moreover, **FS-1** also alleviates the hepatoxicity caused by antibiotics. **SMARt-420** alters EthA-dependent drug-bioactivation pathways restoring susceptibility in ETH-resistant pathogens.

There is an urgent need for developing a drug discovery pipeline against NTM infections. The in vitro assays must include SGM and RGM species along with *M. tb* as part of the screening. Available data suggest that TB inactive compounds may retain activity against NTM species, encouraging a broader perspective on potential candidates for treating NTM infections. In testing NTM species, different morphotypes should be assessed because of the potential for varied susceptibility to antimicrobials [26,98].

In conclusion, MmpL3 inhibitors have potent activity against SGM and RGM. Studies also report synergistic action of MmpL3 inhibitors with TB antibiotics in vitro and in vivo [99,100]. As treatment of NTM infections involves multidrug regimens, adding an MmpL3 inhibitor to the current therapeutic regimen may be beneficial. Therefore, more research is required to explore MmpL3 inhibitors against NTM species and to develop a constant pipeline of drug candidates for treatment of NTM infections.

## Figures and Tables

**Table 1:** Active MmpL3 inhibitors against *M. abscessus*.

Compound Number	Structure	MIC	Ref.
**1**	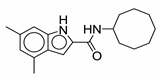	*M. abscessus* = 0.063 µg/mL	[56]
		*M. bolletti* = 0.039 µg/mL	
		*M. massiliense* = 0.031 µg/mL	
		*M. chelonae* = 0.063 µg/mL	
		*M. avium* = 0.05–1 µg/mL	
		*M. xenopi* = 0.25 µg/mL	
**2**	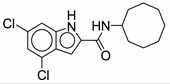	*M. abscessus* = 0.125 µg/mL	[62]
**3**	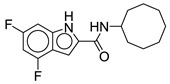	*M. abscessus* = 0.125 µg/mL	[62]
**4**	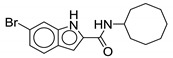	*M. abscessus* = 0.125 µg/mL	[62]
**5**	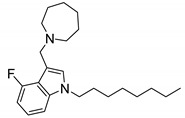	*M. avium* = 2 µM	[63]
		*M. abscessus* = 19 µM	
**6**	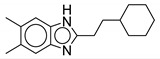	*M. abscessus* = 0.125 µg/mL	[68]
**7**	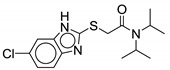	M. abscessus MIC_50_ = 25 µM	[48]
**8**	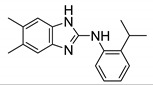	*M. abscessus* MIC_90_ = 31.25 μM	[69]
**9**	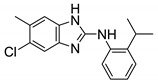	*M. abscessus* MIC_90_ = 31.25 μM	[69]
**10**	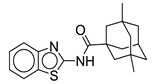	*M. abscessus* = 1 µg/mL	[70]
		*M. avium* = 2 µg/mL	
**11**	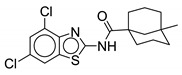	*M. abscessus* = 0.03 µg/mL	[70]
		*M. peregrinum* = 0.03 µg/mL	
		*M. massiliense* = 0.03 µg/mL	
		*M. fortuitum* = 0.03 µg/mL	
		*M. chelonae* = 0.03 µg/mL	
		*M. avium* = 2 µg/mL	
		*M. intracellulare* = 2 µg/mL	
**12**	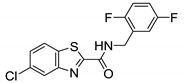	N.D.	[72]
**13**	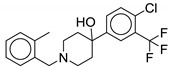	*M. abscessus* MIC_99_ = 0.125 µg/mL	[46,47,48,49,50,51,52,53,54,55,56,57,58,59,60,61,62,63,64,65,66,67,68,69,70,71,72,73,74]
		*M. avium* MIC_90_ > 50 µM	
**14**	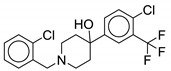	*M. abscessus* MIC_99_ = 0.125 µg/mL	[75]
**15**	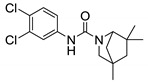	*M. abscessus* MIC_90_ > 62.5 µM	[69]
**16**	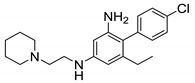	*M. abscessus* MIC_90_ > 31.25 µM	[69]
**17**	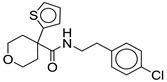	*M. abscessus* MIC_50_ = 6.25 µM	[76]
**18**	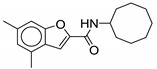	*M. abscessus* = 0.5 µg/mL	[62]
**19**	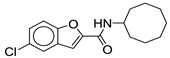	*M. abscessus* = 0.5 µg/mL	[62]
**20**	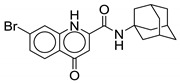	*M. abscessus* > 159 µM	[60]
		*M. avium* > 159 µM	
**21**	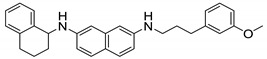	*M. abscessus* MIC_90_ > 31.25 µM	[69]
**22**	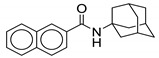	*M. abscessus* > 209 µM	[60]
		*M. avium* > 209 µM	
**23**	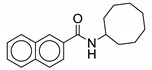	*M. abscessus* > 209 µM	[60]
		*M. avium* > 209 µM	
**24**	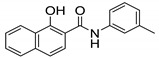	*M. marinum* and *M. kansasii* = 28.8 µM	[77]
**25**	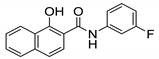	*M. marinum* and *M. kansasii* = 28.4 µM	[77]
**26**	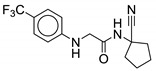	*M. abscessus* MIC_90_ = 12 µM	[49]
		*M. avium* MIC_90_ > 100 µM	
**27**	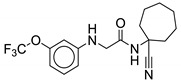	N.D.	[49]
**28**	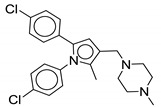	*M. avium* = 2 µM	[79,80]
**29**	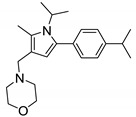	N.D.	[79,80]
